# Construction of monocyte-related prognosis model based on comprehensive analysis of bulk RNA-seq and single-cell RNA-seq in high-grade serous ovarian cancer

**DOI:** 10.1097/MD.0000000000036548

**Published:** 2023-12-15

**Authors:** Ye Xu, Shu Tan, Wei Huang, Yao-Xian Wang

**Affiliations:** a Gynecological Ward, Harbin Medical University Cancer Hospital, Harbin, Heilongjiang, China.

**Keywords:** bulk RNA, high-grade serous ovarian cancer, monocyte, prognosis, single-cell RNA

## Abstract

High-grade serous ovarian cancer (HGSOC) is a common subtype of ovarian cancer with high mortality. Finding a new biomarker is useful for the diagnosis and treatment of HGSOC. The scRNA and bulk RNA data were obtained from The Cancer Genome Atlas and Gene Expression Omnibus databases. The monocyte-related clusters were identified and annotated by Seruat and SingleR package. The Kaplan–Meier and receiver operating characteristic curve was used to determine the prognosis. The differentially expressed genes were determined by limma. The single sample Gene Set Enrichment Analysis, Gene Set Enrichment Analysis, Gene Ontology, and Kyoto Encyclopedia of Genes and Genomes were used for the enrichment function. The correlation between drug activity and gene expression was assessed by rcellminer and rcellminer Data package. We identified 9 cell types and obtained 37 differentially expressed marker genes of monocyte. A2M, CD163, and FPR1 were screened out as hub genes and used to construct risk model in HGSOC through univariate and multivariate cox analysis. Single sample Gene Set Enrichment Analysis showed risk score was related to B cell and T cell signal pathways, and further analysis showed most immune checkpoint genes expressions were upregulated in high-risk score group. The Gene Ontology and Kyoto Encyclopedia of Genes and Genomes analysis exhibited that hub gene related genes were involved in signal receptor binding and cytokine-cytokine interaction. Low A2M expression and high expression of CD163 and FPR1 were associated with poor prognosis. Gene Set Enrichment Analysis revealed that A2M promoted tumor development through enhancing immune cell related signal pathways, while CD163 and FPR1 inhibited tumor development through activated carcinogenic signal pathways. Drug sensitivity analysis revealed that these hub genes could be potential therapeutic targets for the treatment of HGSOC. We constructed a risk model for the overall survival and explored the potential mechanism of monocyte in HGSOC.

## 1. Introduction

Ovarian cancer (OC) is a prevalent malignancy among women, ranking seventh worldwide.^[1]^ The majority of malignant ovarian tumors are epithelial ovarian cancer and high-grade serous ovarian cancer (HGSOC) accounts for most epithelial ovarian cancer.^[2]^ Previous studies showed that patients diagnosed with HGSOC in the early stage have a favorable prognosis.^[3]^ However, because of the asymptomatic nature of HGSOC and inadequacies of the test method, most patients were diagnosed in the advanced stage,^[4]^ and the 5-year survival rate of them was <40%.^[5]^ Until now, surgery and chemotherapy were the main methods for the first-line treatment of HGSOC, but the survival rate of HGSOC is not improved.^[6]^ Some clinical results identified that the incorporation of targeted anti-PD-1 treatment with pegylated liposomal doxorubicin into first-line management of ovarian cancer can improve survival.^[7,8]^ Besides, for OC patients with recurrence after first-line treatment, an antiangiogenic treatment combine with the chemotherapy backbone was usually applied.^[9]^ Recently, an anti-poly adenosine diphosphate-ribose polymerase maintenance treatment (Olaparib) is approved for OC patients with recurrence, which has achieved clinical improvements.^[10]^ However, for most patients with OC, there is still a crucial need to develop new therapies.

Monocyte is a major part of the innate immune system which has a vital function in immune response.^[11]^ The subsets of monocytes contain classical, nonclassical, and intermediate monocytes.^[12]^ Monocytes are first differentiated into classical monocytes in the bone marrow, then classical monocytes are differentiated into nonclassical monocytes in the blood, and there is a transition state called intermediate monocytes in this progression.^[13]^

Numerous studies have provided evidence indicating that monocytes play a direct role in the immune response through inducing cell death and phagocytosis.^[14,15]^ Additionally, monocytes have the ability to interact with T and natural killer cells, thereby influencing tumor development through inducing the production of chemokines.^[16,17]^ Besides, monocytes differentiate into various immune cells, containing tumor-related macrophages and DCs, which are integral components of the immune system and actively contribute to tumor development and metastasis.^[18]^ Despite typically having a tumoricidal role in the majority of cancers, the specific cancer types, issues, and tumor micro environment could cause the contrasting function of monocytes in tumor development.^[18]^ To date, monocytes showed encouraging value in the prediction of prognosis in multiple cancers. Moreover, studies have indicated that the lymphocyte-monocyte ratio may serve as an independent prognostic factor for OC.^[19]^ However, there is currently a lack of research on the monocyte-related risk model in HGSOC.

Therefore, in this study, we identified the monocyte-related genes through scRNA-seq and bulk RNA-seq, following the construction of a risk model. Besides, we made preliminary research on the mechanism of the monocyte-related genes in HGSOC.

## 2. Methods

### 2.1. Data collection

The bulk RNA-seq, scRNA-seq (GSE154600), and clinical information of OC were obtained from The Cancer Genome Atlas (TCGA, https://portal.gdc.cancer.gov) and Gene Expression Omnibus (GEO, https://www.ncbi.nlm.nih.gov/geo/) databases. The patients diagnosed with stages II to IV were considered as HGSOC.^[20]^ Besides, 79 immune checkpoint genes were determined through the previous literature.^[21]^

### 2.2. scRNA-seq data analysis

At first, the Seurat package was used for the quality control of scRNA-seq. The standard was set as follows: the number of the expressed gene was >500, and the total number of detected molecules >1000 or <20,000, and the content of mitochondria was <10%. Then, after the data were normalized, the top 2000 highly variable genes were identified using the Find Variable Features function. Next, principal component analysis was applied to the dimensionality reduction, and ElbowPlot was used to determine the principal component. Subsequently, the cell was clustered by the t-SNE algorithm, and the marker genes of every cluster were identified by the FindAllMarkers function. Finally, the cell subtypes were annotated using the SingleR package in accordance with maker genes.

### 2.3. Survival analysis

The Kaplan–Meier curve was used to evaluate the difference of overall survival between different groups. After we determined the optimal truncation value, the samples were divided into 2 groups. Then we assessed the difference of prognosis between the 2 groups by the survfit function of the survival package.

### 2.4. The construction and validation of the risk model

The limma package was utilized for the selection of differentially expressed analysis. Genes with |log2 FC| > 1 and *P* value < .05 were considered as differentially expressed genes (DEGs). Then, 20 genes were screened out in the intersection of DEGs and mark genes. The hub genes were identified using univariate and multivariate cox analysis with *P* < .05 based on the 20 genes. Next, we constructed the risk model and calculated the risk score in accordance with the gene expression and regression coefficient. Besides, the area under the curve for 1-, 3-, and 5-year survival was evaluated by the pROC package, which could assess the predictive efficiency of the risk model.

### 2.5. Single sample Gene Set Enrichment Analysis

The geneset (C2: KEGG subset of CP) was downloaded from the Molecular Signatures database (https://www.gsea-msigdb.org/gsea/msigdb/human/collections.jsp#C2). After the expression and phenotype profiles were imported into R software, the GSVA package was used for the evaluation of the enrichment score. Besides, the correlation between the scores and signal pathways was calculated by Pearson method.

### 2.6. The construction of PPI network

We first constructed a PPI network using STRING. The cutoff value of the interaction score was 0.4, and the number of 1st shell and 2nd shell was no more than 50 interactors. Then, the cytoHubba was used for the extraction of the top 15 genes.

### 2.7. Gene Ontology (GO) and Kyoto Encyclopedia of Genes and Genomes (KEGG) enrichment analysis

After the genes related to hub genes were determined in STRING, the GO and KEGG enrichment analyses were performed. The annotation files (c2.cp.kegg.v7.4.symbols and c5.go.v7.4.symbols) were obtained from GitHub. After the gene expression profile and annotation files were loaded, the cluster Profiler package was executed for the GO and KEGG analysis. The minimum gene was set to 5 and the maximum gene was set to 5000. The *P* value < 0.05 was considered statistically significant.

### 2.8. Gene Set Enrichment Analysis (GSEA) analysis

Then, the GSEA enrichment analysis was performed in GSEA software (version 4.2.3) to elucidate the function of hub genes. The gene set “c2.cp.kegg.v2023.1.Hs.symbols” was selected for further study. The number of permutations was set as 1000, and the enrichment statistic was set as weighted, and the metric for ranking genes was set as Signal2Noise. The max size was 500, and the minimum size was 15.

### 2.9. The correlation between drug activity and gene expression

We tried to determine novel potential targets for the treatment of HGSOC through drug sensitivity analysis. The dataset was downloaded from the CellMiner database (https://discover.nci.nih.gov/cellminer/home.do). The drug results were screened out which were approved by clinical trials and FDA. Next, drug results and expression profile were integrated, and the correlation between drug activity and gene expression was calculated using the corPvalueStudent function. The results were visualized by the ggplot package.

### 2.10. Statistical analysis

In this study, all data were analyzed in SPSS 25 and R studio software. The independent sample *t* test was used to calculate the difference between the 2 groups. The survival difference was evaluated by the log-rank test, and the correlation analysis was identified by the Pearson test. *P* < .05 was considered a significant difference.

## 3. Results

### 3.1. The cell type was identified by scRNA-seq

**Figure 1.**
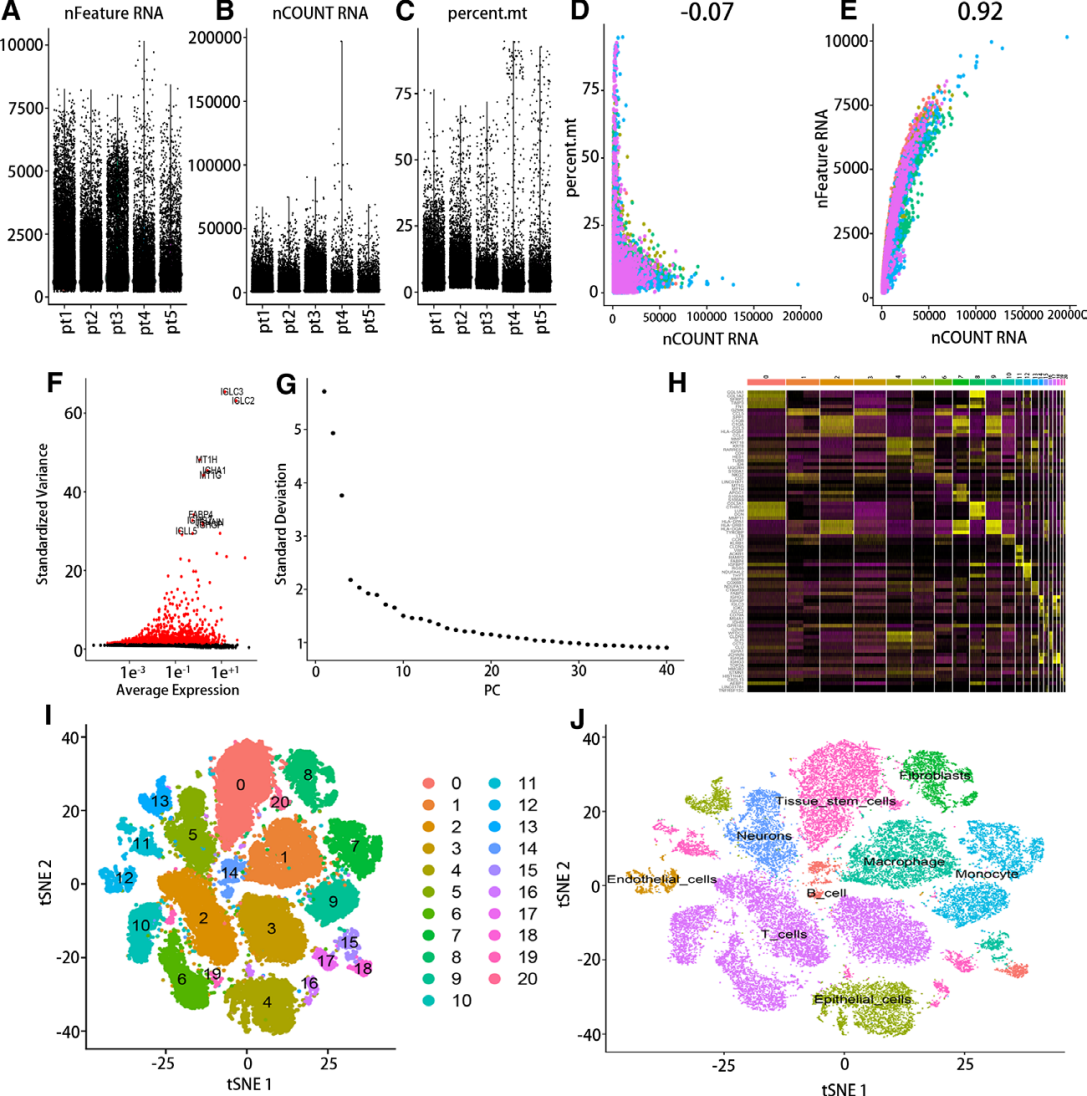
The determination of cell type in HGSOC based on 5 cell samples. (A–C) Quality control of scRNA-seq of 5 HGSOC samples. (D and E) The correlation between sequencing depth and mitochondrial content or the number of genes. (F) The 2000 highly variable genes in HGSOC. (G) The standard deviation of 0 to 40 PCs. (I) 21 clusters were identified using t-SNE algorithm. (J) 9 cell subtype was annotated by SingleR. HGSOC = high-grade serous ovarian cancer.

In this study, 51,643 cells of 5 HGSOC samples were used for the scRNA-seq. The depth and width of detection were shown in Figure 1A and B, and the percent of mitochondrial content was exhibited in Figure 1C. Additionally, there was no correlation between the percent of mitochondrial and RNA count (Fig. 1D), while RNA count was closely and positively related to the number of genes (Fig. 1E). After the expressions were normalized, the 2000 highly variable genes were selected and top 10 genes were exhibited in Figure 1F. Furthermore, 13 PCs were determined through principal component analysis (Fig. 1G). Besides, the top 5 marker genes in every cluster were presented in the heatmap (Fig. 1H). Then, all cells were separated into 21 clusters through the t-SNE algorithm, and cell subpopulations were annotated by SingleR (Figs. 1I and J). The cluster 0, 11, 16, 17, and 20 were tissue stem cells, cluster 1 and 15 were macrophage, cluster 2, 3, 6, 10, and 19 were T cells, cluster 4 and 13 were epithelial cells, cluster 5 was neurons, cluster 7 and 9 were monocyte, cluster 8 was fibroblasts, cluster 12 was endothelial cells, and cluster 14 and 18 were B cells.

### 3.2. The establishment and validation of the monocyte-related risk model

**Figure 2.**
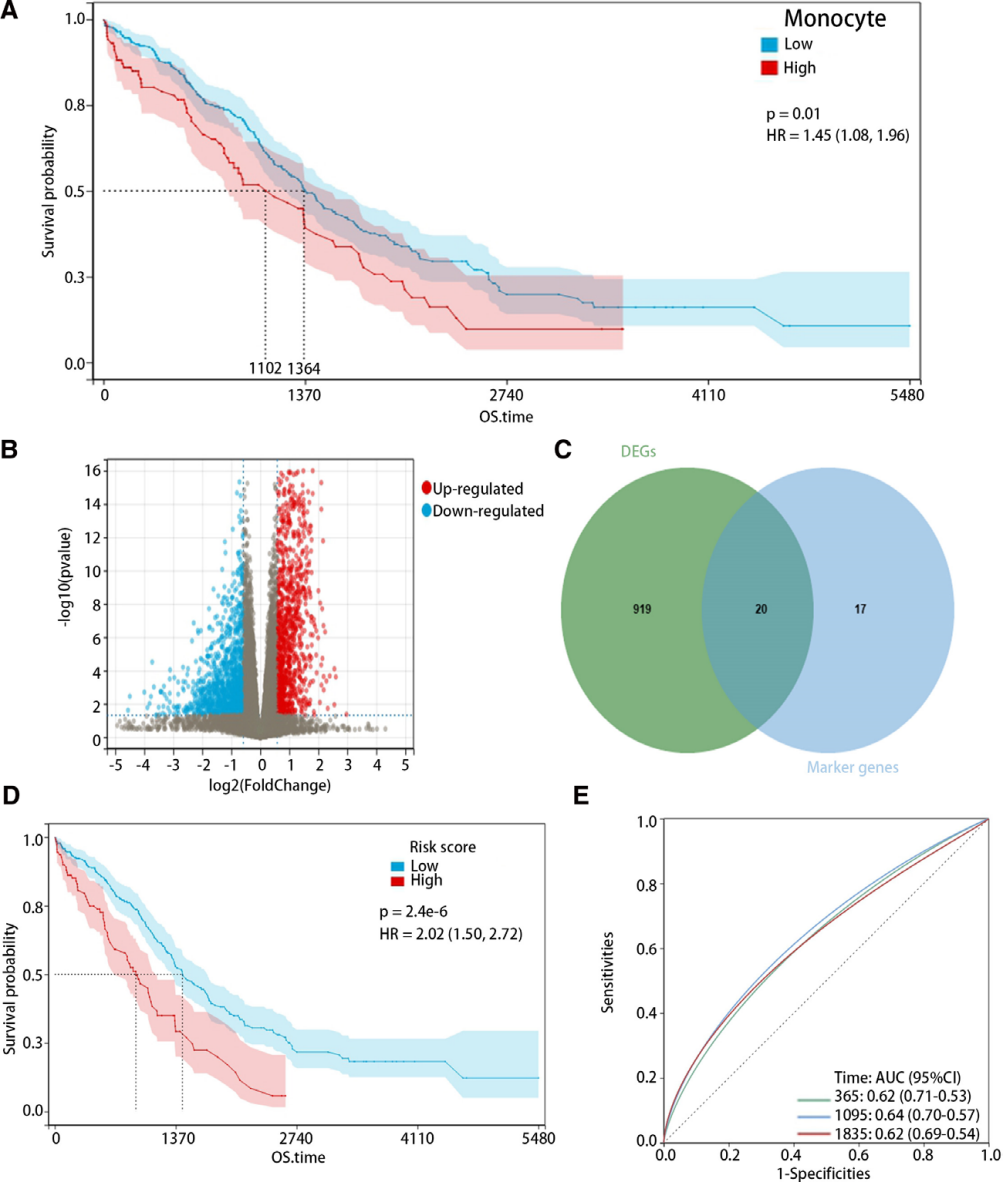
The monocyte-related hub genes were screened out and used to construct a risk model. (A) The K-M curve of patients with different immune scores of monocytes. (B) The volcano plot of DEGs in high and low immune scores of monocytes. (C) The intersection of DEGs and marker genes. (D) The K-M plot of patients with high and low risk scores. (E) The ROC curve for 1-, 3-, and 5-year survival. DEGs = differentially expressed genes, K-M = Kaplan–Meier, ROC = receiver operating characteristic.

After the immune infiltration scores of monocyte cells were calculated in HGSOC through MCP, we proceeded to evaluate the impact of monocytes on overall survival time. The findings indicated a significant association between the high immune infiltration level of monocytes and poor prognosis (Fig. 2A), and the DEGs between high and low groups were identified (Fig. 2B). After the intersection of DEGs and marker genes were determined (Fig. 2C), the hub genes were screened out through univariate and multivariate cox regression analysis (Table 1). According to the regression coefficient, we constructed a risk model as follows: risk score = −0.07 × exp(A2M) + 0.3 × exp(CD163) + 0.63 × exp(FPR1). The survival analysis exhibited that the high risk score was obviously associated with a poor prognosis (Fig. 2D). Moreover, the receiver operating characteristic curve showed the area under the curve for 1-, 3-, and 5-year survival time as 0.62, 0.64 and 0.62, respectively (Fig. 2E).

**Table 1:** Univariate and multivariate Cox regression analysis of 6 genes.

	Univariate		Multivariate	
	HR (95% CI)	*P* value	HR (95% CI)	*P* value
FPR1	1.09	1.4e−4	1.09	0.02
CD163	1.03	1.9e−4	1.04	0.02
CD14	1.00	0.01	1.00	0.26
ITGAM	1.09	0.02	1.03	0.68
ABCA1	1.06	0.04	1.03	0.43
A2M	1.00	0.04	0.99	1.2e−3

CI = confidence interval, HR = hazard ratio.

### 3.3. The role of risk score in signal pathways

**Figure 3.**
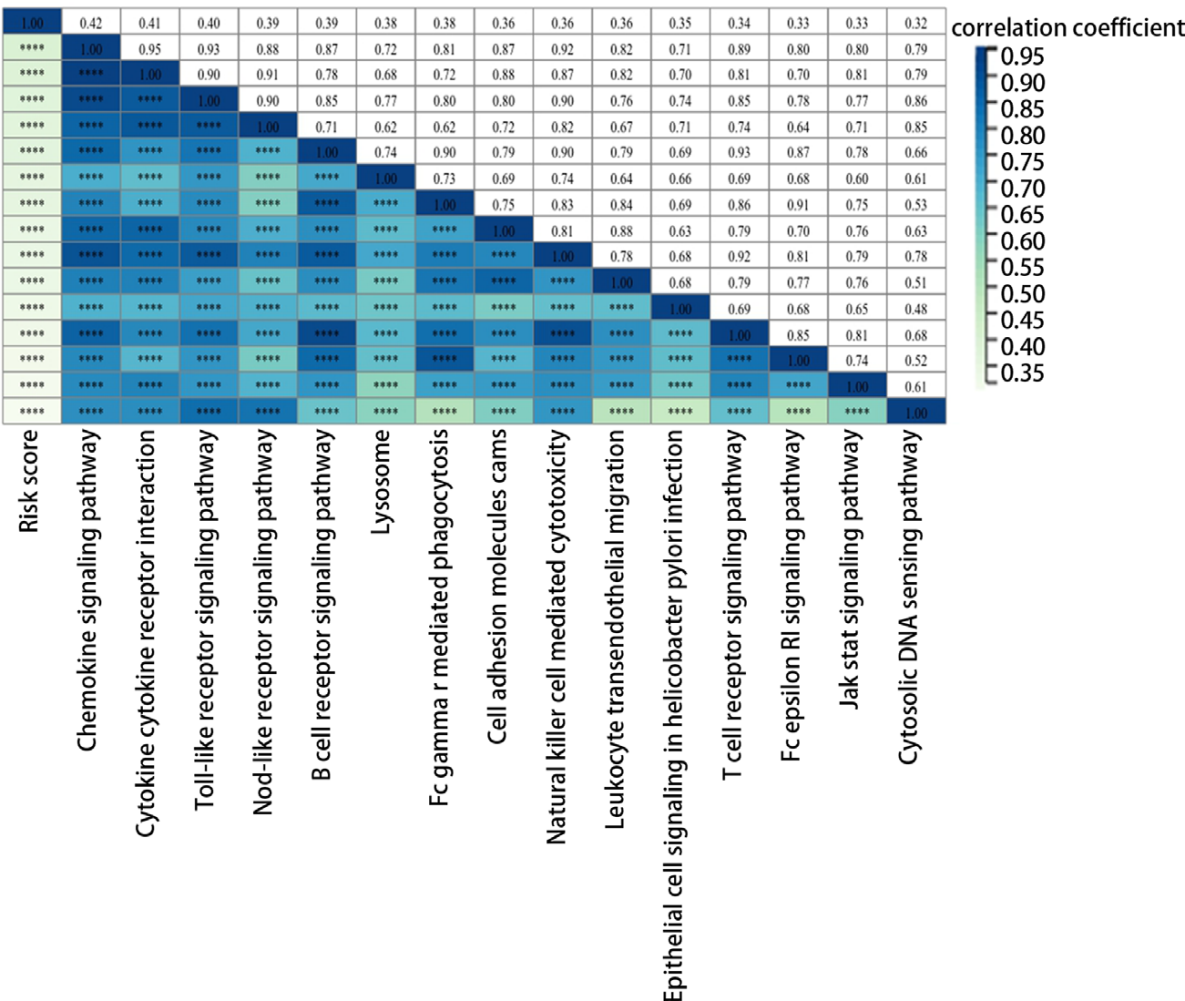
The correlation between risk score and signal pathways.

Next, we further explored the potential mechanism of the risk score in HGSOC through single sample Gene Set Enrichment Analysis. As presented in Figure 3, the risk score was positively and remarkably related to the chemokine signaling pathway, cytokine receptor interaction, and toll-like receptor signaling pathway. Additionally, it was obvious that risk score played a role in B cell and T cell receptor signaling pathways, which had essential functions in the immune process.

### 3.4. The effect of risk score on the expressions of immune check genes (ICGs)

**Figure 4.**
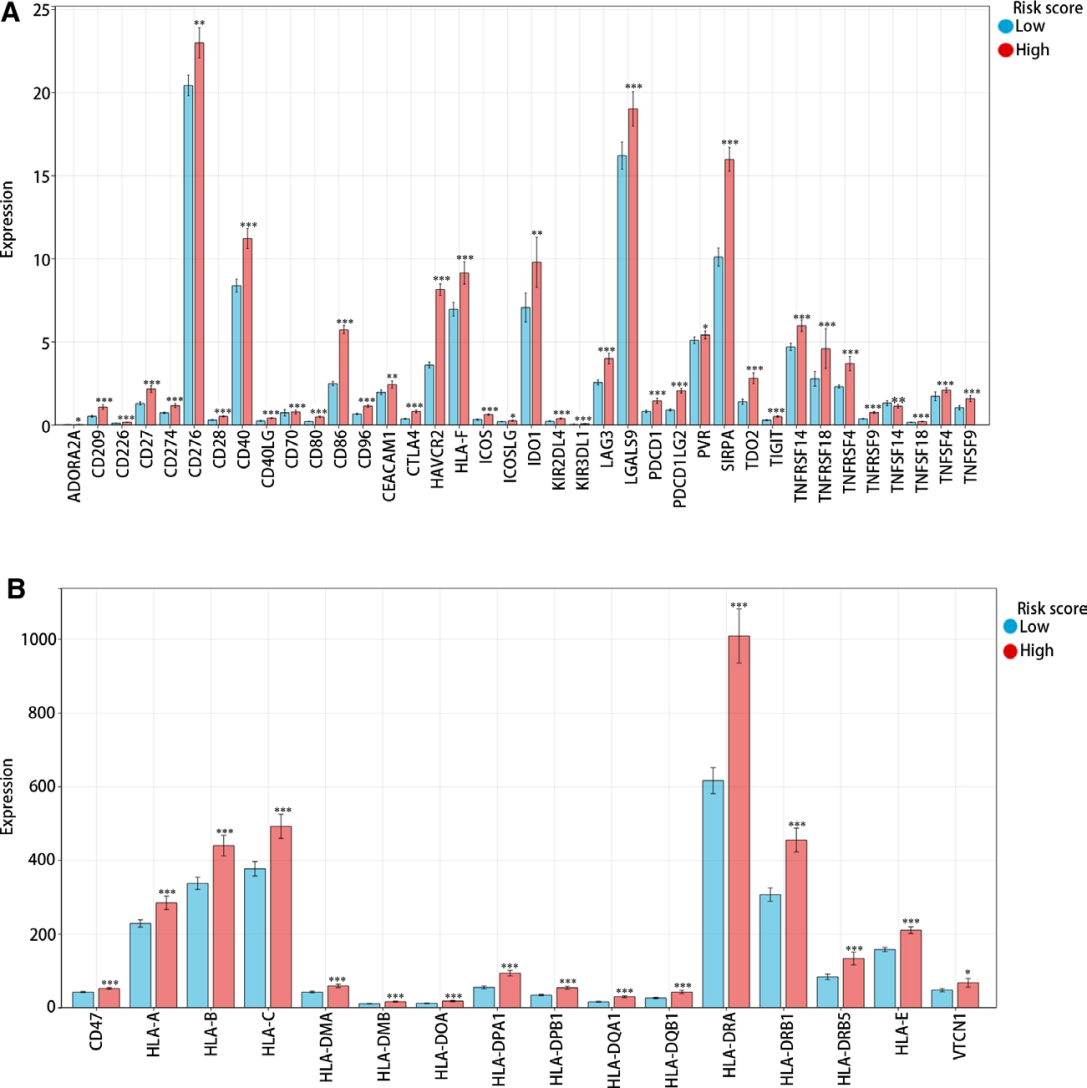
The correlation between risk score and immune checkpoint gene and risk score. (A) The immune checkpoint genes have a low expression level. (B) The immune checkpoint genes have a high expression level. *<.05, **<.01, ***<.001.

Subsequently, we compared the expression of ICGs in different risk score groups. From Figure 4A and B, it could be observed that most ICGs expression was notably higher in the high-risk score group, while the level of TNFSF14 was obviously downgraded.

### 3.5. The construction of hub genes-related PPI network

**Figure 5.**
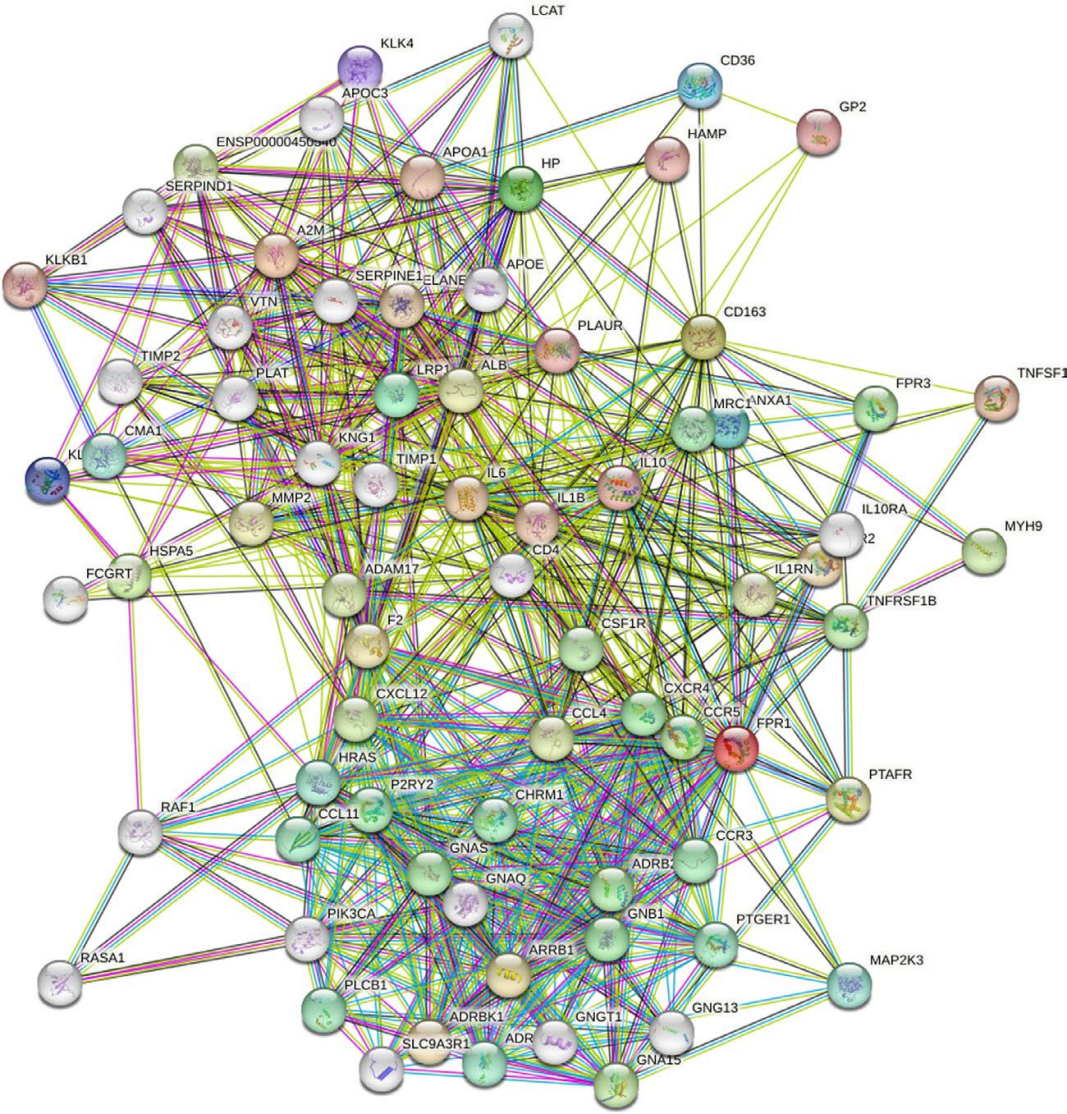
The PPI network was constructed by STRING.

**Figure 6.**
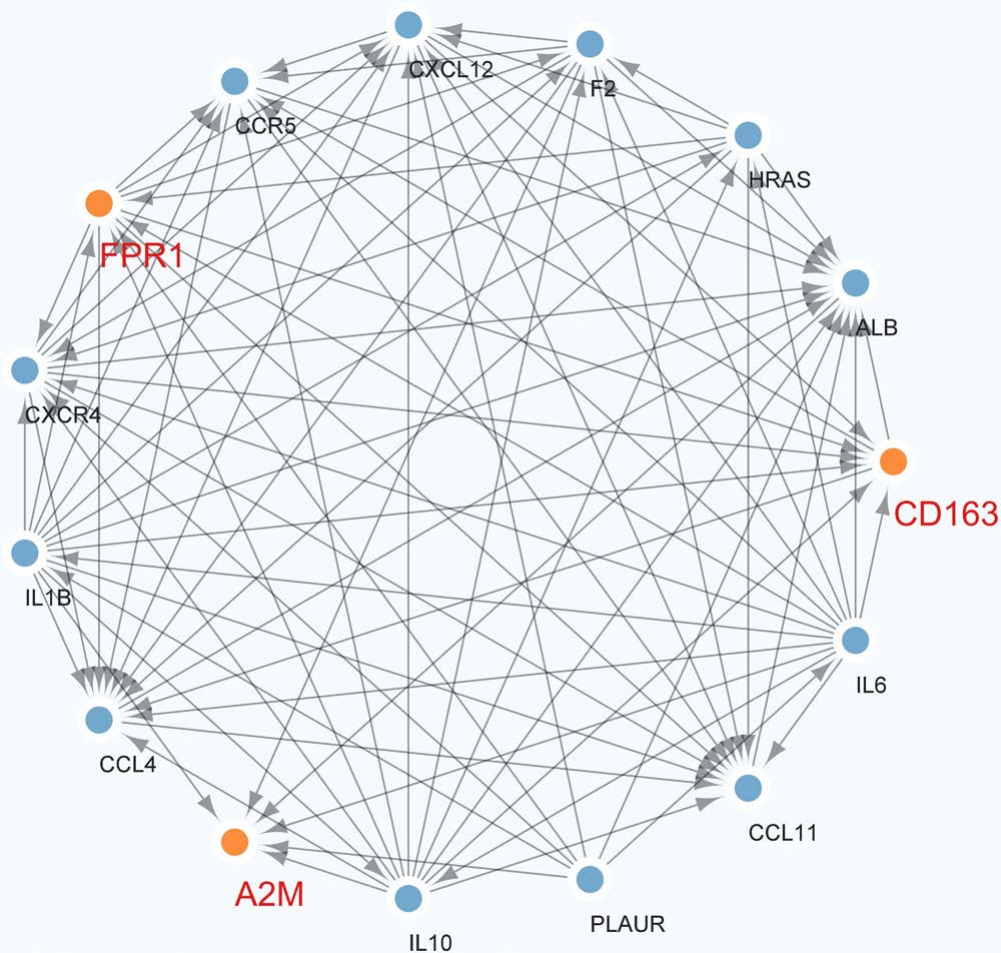
The key subnetwork was extracted from the PPI network.

To further investigate the regulator mechanism of hub genes, we constructed a PPI network based on hub genes using STRING (Fig. 5). In addition, we extracted a vital subnetwork from the PPI network. As can be seen in Figure 6, there was a correlation between CD163 and FPR1, but A2M could not directly interact with CD163 or FPR1.

### 3.6. The GO and KEGG enrichment analysis

**Figure 7.**
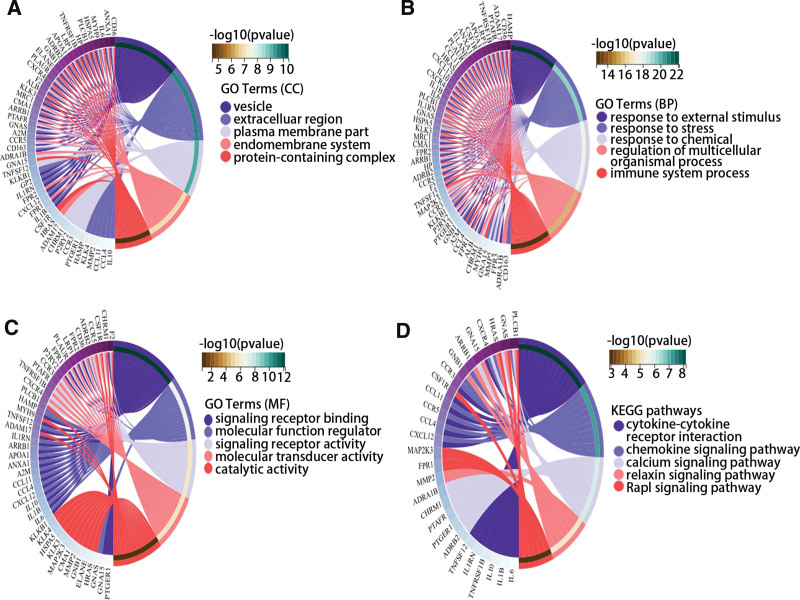
The function enrichment of hub gene-related genes was analyzed by (A–C) GO and (D) KEGG. GO = Gene Ontology, KEGG = Kyoto Encyclopedia of Genes and Genomes.

The GO and KEGG analyses were used to determine the function of genes obtained from the PPI network. In the aspect of CC, these genes were enriched in vesicle, extracellular region, plasma membrane part, endomembrane system, and protein-containing complex (Fig. 7A). The BP of these genes included response to external stimulus, response to stress, response to chemical, regulation of multicellular organismal process (Fig. 7B). In the fields of MF, these genes were enriched in signaling receptor binding, molecular function regulator, signaling receptor activity, molecular transducer activity, and catalytic activity (Fig. 7C). Besides, KEGG results showed that these genes were mainly involved in the cytokine-cytokine receptor interaction, chemokine signaling pathway, calcium signaling pathway, relaxin signaling pathway, and Rap1 signaling pathway (Fig. 7D).

### 3.7. The expression and prognosis of hub genes in HGSOC

**Figure 8.**
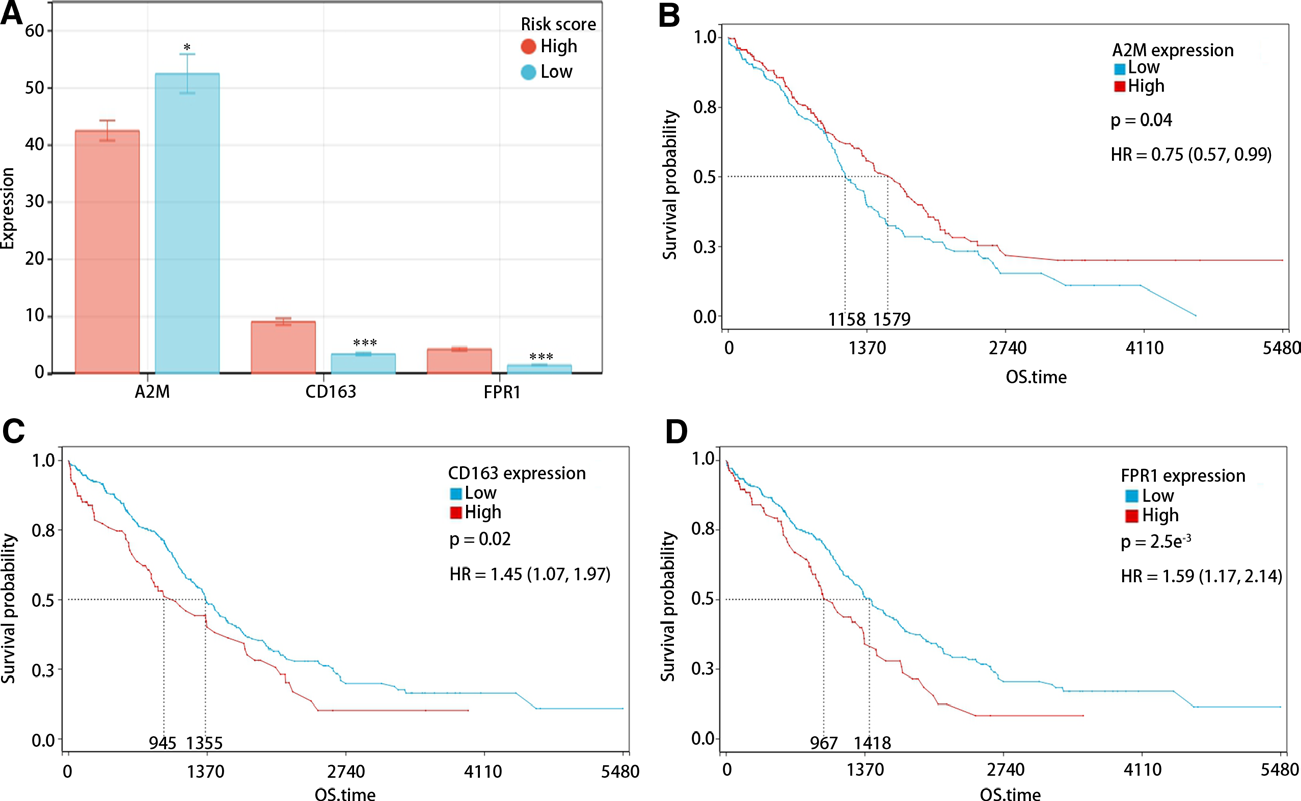
The value of hub genes expression in risk score and prognosis. (A) The expression of hub genes in high and low risk score groups. (B–D) K-M curve of (B) A2M, (C) CD163, and (D) FPR1 expressions. K-M = Kaplan–Meier.

Figure 8A demonstrated a significant downregulation of CD163 and FPR1 expressions in the low-risk score group in comparison with the high-risk score group, while A2M expression was upregulated. In addition, survival analysis revealed a strong association between low A2M expression and poor prognosis, and high expression of CD163 and FPR1 were closely related to the poor prognosis (Fig. 8B and D).

### 3.8. The GSEA enrichment analysis of hub genes in HGSOC

**Figure 9.**
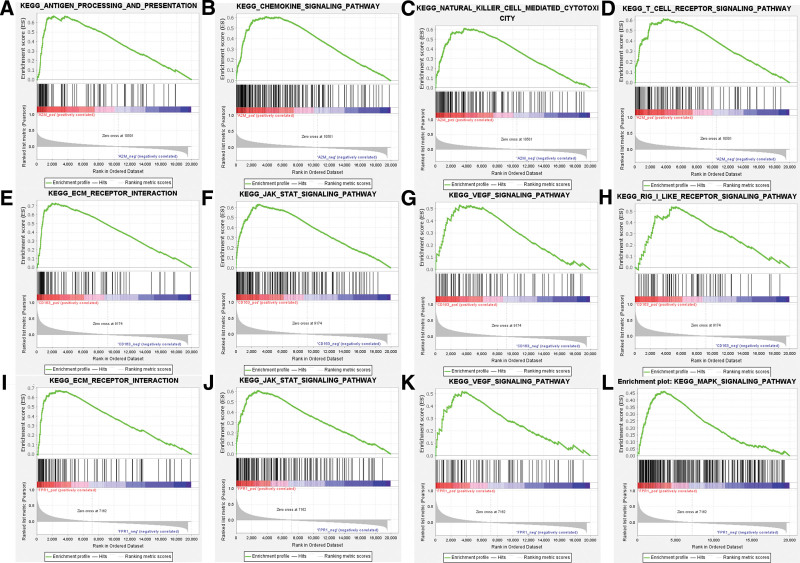
The function enrichment of (A–D) A2M, (E–H) CD163, and (I–L) FPR1 in HGSOC. HGSOC = high-grade serous ovarian cancer.

The GSEA results were presented in Figure 9. It displayed that the function of A2M was enriched in antigen processing and presentation, chemokine signaling pathway, natural cell mediated cytotoxicity, and T cell receptor signaling pathway, which were involved in the suppression of tumor development (Fig. 9A–D). Besides, CD163 and FPR1 both play a role in the ECM receptor interaction, JAK STAT signaling pathway, and VEGF signaling pathway (Fig. 9E–G and I–K). Furthermore, CD163 and FPR1 were a role factor of RIG-I like receptor and MAPK signaling pathway, respectively (Fig. 9H and L).

### 3.9. The role of hub genes expressions in drug activity

**Figure 10.**
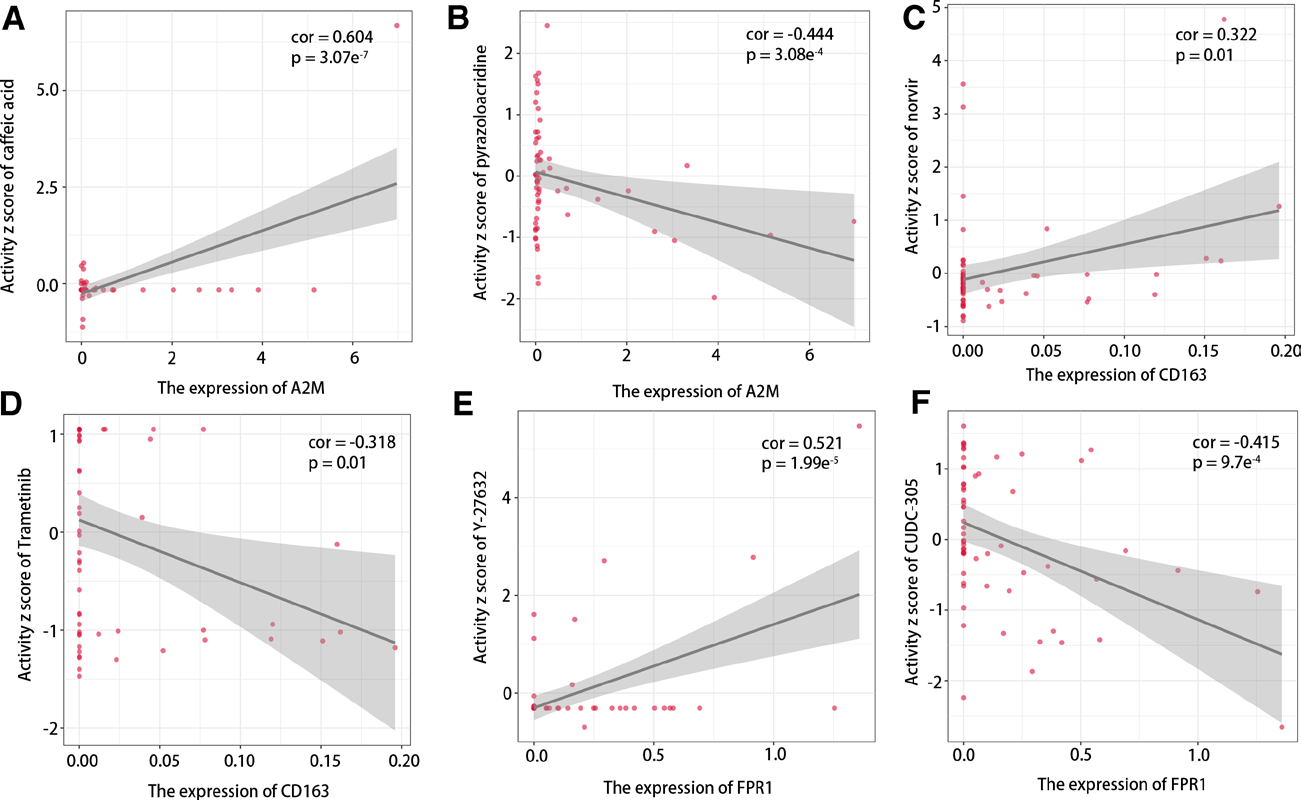
The correlation between drug activity and the expression of hub genes including (A and B) A2M, (C and D) CD163, and (E and F) FPR1.

Finally, we assessed the clinical value of hub genes. The results showed 47 drugs were memorably related to hub genes, and the highest positive and negative correlations between drug activity and hub gene expressions were displayed in Figure 10. It could be seen that A2M, CD163, and FPR1 were positively related to caffeic acid, norvir, and Y-27632, respectively, while they were negatively related to pyrazoloacridine, trametinib, and CUDC-305.

## 4. Discussion

In this study, a total of 21 clusters were identified and 9 cell types were annotated using scRNA-seq, utilizing data from 5 patients diagnosed with HGSOC obtained from the GEO database. Previous studies have highlighted the crucial role of monocytes in the tumor microenvironment, as they play a pivotal role in tumor growth and development.^[22]^ Additionally, the dysfunction of monocytes has been implicated in the development of primary immunodeficiency diseases which promoted the development of tumor.^[23]^ However, it was important to note that the function of monocytes was affected by various factors such as the tumor sites, stages, and other factors, leading to divergent roles in different types of cancer.^[18]^ Franklin et al^[24]^ found that classical monocytes were differentiated into tumor-related macrophages with the purpose of inhibiting the activity of cytotoxic T cells within mammary tumors, which promoted the development of tumors. Similarly, nonclassical monocytes also exhibited a positive function in the process of oncogenesis through promoting angiogenesis and suppressing the activity of T cells.^[25,26]^ On the contrary, both classical and nonclassical monocytes were found to possess the ability to impede the development of tumor cells through the induction of apoptosis and recruitment of natural killer cells.^[27,28]^

According to our results, it was determined that a high level of monocyte immune level was remarkably associated with an unfavorable prognosis, which was consistent with previous studies.^[29]^ It suggested that monocytes may have a detrimental impact on the development of HGSOC. Then, a risk model was developed based on the expression of 20 monocyte-related genes, revealing a negative correlation between the calculated risk score and prognosis. Furthermore, function enrichment analysis demonstrated a clear and positive association between these genes and multiple signal pathways, such as toll-like receptor signaling pathway, B cell receptor signaling pathway, and T cell signaling pathway. The findings suggested that monocytes were involved in tumor development through modulating immune response and activating carcinogenesis-related signal pathways. The further results indicated that most ICGs expressions were upgraded in the high-risk score group. Additionally, enrichment analysis revealed that the genes, which had a tight association with hub genes, were enriched in processes related to the immune system progress and signaling receptor binding. These findings provide evidence that monocytes may affect the development of HGSOC through regulating the immune-related signal factors. Previous studies have established that ICGs could possess the capability to hinder the activity of immune cells, thereby facilitating the evasion of tumor cells from immune surveillance.^[30–32]^ Our investigation revealed that the majority of ICGs expression exhibited increased expression in the high-risk score group. This finding suggested that monocytes may exert an effect on immune cells through interacting with immune checkpoints, consequently facilitating immune escape.

A2M, functioning as a proteinase inhibitor, impedes proteolysis and elicits immune responses.^[33,34]^ By binding to cytokines and growth factors such as IL-4, TGF-beta1, and VEGF, A2M can effectively impede tumor development.^[35–37]^ Additionally, the augmentation of antigen presentation in macrophages by A2M may lead to an enhanced immune response.^[38]^

CD163, a monocyte/macrophage related scavenger receptor,^[39]^ has been utilized to differentiate M1 and M2 macrophages.^[40]^ Moreover, CD163 has been used as a potential biomarker for multiple cancers. Most studies identified that high expression of CD163 was closely associated with poor prognosis in multiple cancers,^[41,42]^ but Pelekanou et al^[43]^ demonstrated that high levels of CD163 may be related to favorable prognosis in breast cancer.

FPR1, a G protein-coupled receptor, has been implicated in both innate and adaptive immunity.^[44,45]^ Vecchi et al^[46]^ conducted a study demonstrating the inhibitory effects of FPR1 inhibitors on the growth and metastasis of triple-negative breast cancer. In addition, Jiang et al^[47]^ found that FPR1 played a negative role in the development of tumors through promoting the cell cycles. These results indicated that FPR1 may be an oncogene. Besides, previous studies have elucidated that FPR1 promoted the development of tumors through inducing inflammation-related signal pathways,^[48–50]^ and its association with unfavorable prognosis has been observed in diverse malignancies.^[51]^

In our study, we found low expression of A2M was closely related to poor prognosis in HGSOC, while high expressions of CD163 and FPR1 were closely related to poor prognosis. The further results showed that A2M may repress the development of HGSOC through enhancing antigen presentation to activate immune cells. Furthermore, both CD163 and FPR1 played a vital and positive role in the tumor-promoting signal pathways in order to promote the development of HGSOC. Although FPR1 upregulated the expression of resistance-related genes,^[47]^ we found some drug activity was positively related to FPR1 expression, especially Y-27632. It meant some drugs may be potential targets for the treatment of HGSOC.

There are some limitations in our study. First, the data used in our study were only obtained from TCGA and GEO, and it need to be validated in more prospective datasets to avoid the potential risks of error and deviation. Second, the most patients in TCGA were white. Thus, it remains unclear whether this result could be used for other races. Third, the results were analyzed using bioinformation, and the function of hub genes and the potential mechanism in OV should be identified through experiments in vivo and in vitro. However, our study still provides a new sight and some basic theoretical basis for exploring the mechanism of OV development. Moreover, with the improvement of sequencing accuracy and the increasing amount of data in public databases, we will be able to obtain more accurate prediction results through bioinformatics, which will greatly reduce economic and time costs.

In conclusion, we constructed a risk model based on 3 hub genes in HGSOC which were screened out from the intersection of the marker genes of monocyte and DEGs. The bioinformatics analysis showed monocytes may be involved in the development of HGSOC through enhancing immune response.

## Author contributions

**Conceptualization:** Ye Xu, Shu Tan.

**Data curation:** Ye Xu, Shu Tan, Wei Huang, Yao-Xian Wang.

**Formal analysis:** Ye Xu, Shu Tan, Wei Huang, Yao-Xian Wang.

**Methodology:** Yao-Xian Wang.

**Writing – original draft:** Shu Tan.

**Writing – review & editing:** Wei Huang.
